# Development and validation of a novel nomogram for predicting recurrent atrial fibrillation after cryoballoon ablation

**DOI:** 10.3389/fcvm.2023.1073108

**Published:** 2023-08-11

**Authors:** Yue Wei, Changjian Lin, Yun Xie, Yangyang Bao, Qingzhi Luo, Ning Zhang, Liqun Wu

**Affiliations:** Department of Cardiovascular Medicine, Ruijin Hospital, Shanghai Jiao Tong University School of Medicine, Shanghai, China

**Keywords:** atrial fibrillation, cryoballoon ablation, nomogram, prediction model, post-ablation recurrence

## Abstract

**Background:**

Few studies have explored the use of machine learning models to predict the recurrence of atrial fibrillation (AF) in patients who have undergone cryoballoon ablation (CBA). We aimed to explore the risk factors for the recurrence of AF after CBA in order to construct a nomogram that could predict this risk.

**Methods:**

Data of 498 patients who had undergone CBA at Ruijin Hospital, Shanghai Jiaotong University School of Medicine, were retrospectively collected. Factors such as clinical characteristics and biophysical parameters during the CBA procedure were collected for the selection of variables. Scores for all the biophysical factors—such as time to pulmonary vein isolation (TTI) and balloon temperature—were calculated to enable construction of the model, which was then calibrated and compared with the risk scores.

**Results:**

A 36-month follow-up showed that 177 (35.5%) of the 489 patients experienced AF recurrence. The left atrial volume, TTI, nadir cryoballoon temperature, and number of unsuccessful freezes were related to the recurrence of AF (*P* < .05). The area under the curve (AUC) of the nomogram's time-dependent receiver operating characteristic curve was 77.6%, 71.6%, and 71.0%, respectively, for the 1-, 2-, and 3-year prediction of recurrence in the training cohort and 77.4%, 74.7%, and 68.7%, respectively, for the same characteristics in the validation cohort. Calibration and data on the nomogram's clinical effectiveness showed it to be accurate for the prediction of recurrence in both the training and validation cohorts as compared with established risk scores.

**Conclusion:**

Biophysical parameters such as TTI and cryoballoon temperature have a great impact on AF recurrence. The predictive accuracy for recurrence of our nomogram was superior to that of conventional risk scores.

## Introduction

Pulmonary vein isolation (PVI) is the cornerstone of efforts to ablate atrial fibrillation (AF). Cryoballoon ablation (CBA), designed especially for PVI, is now established as a standard treatment for symptomatic drug-resistant AF. However, there remain some 30% of patients for whom this procedure is ineffective (1). Many scores based on clinical characteristics have been created in efforts to assess the risk of AF recurrence, but their predictive accuracy is limited and variables in some scores are difficult to obtain (2, 3). Biophysical parameters during CBA—such as time to PVI (TTI), balloon temperature, and number of unsuccessful freezes—have been identified as associated with the durability of PVI (4). Accordingly, we speculated that those biophysical parameters were related to the recurrence of AF after CBA and that a predictive model based on them could be more useful than dependence on conventional risk scores.

We therefore aimed to (1) prove that biophysical parameters during CBA could be used as variables in the prediction of AF recurrence, and (2) develop and validate a nomogram for the prediction of recurrence after CBA.

## Methods

### Study population

We recruited 498 consecutive AF patients who had undergone CBA between January 2017 and July 2019 at Ruijin Hospital, Shanghai Jiaotong University School of Medicine. Preoperative cardiac ultrasound and computed tomography angiography (CTA) of left atrium (LA) were performed. The inclusion criteria specified that these patients had to be between the 18 and 80 years of age and experiencing symptoms of AF, in whom PVI by CBA was well indicated and successful PVI was considered as the main endpoint of the procedure, and that they had to have failed or refused a prescription of at least one antiarrhythmic drug (AAD). The exclusion criteria included the following: (1) prior LA ablation, (2) a LA diameter greater than 50 mm, (3) experience of a myocardial infarction with the prior 3 months, (4) a stroke or transient ischemic attack within the prior 6 months, (5) valvular AF, and (6) inability or refusal to accept postinterventional oral anticoagulation (OAC).

### Ablation procedure

Before ablation, AADs with the exception of amiodarone were discontinued for at least 5 half-lives; amiodarone was discontinued for at least 14 days. OAC was continued. Transesophageal echocardiography was required within 3 days of the procedure to assess for a left atrial thrombus. During the procedure, patients were under conscious anesthesia and monitored for their vital signs. Heparin was administered intravenously with a bolus and the activated clotting time (ACT) was monitored and maintained for more than 300 s. A decapolar catheter was placed in the coronary sinus, with a duopolar catheter in the right ventricular apex for backup pacing. The LA was accessed with a steerable sheath (FlexCath Advance, Medtronic, Minneapolis, MN, USA), through which the CB (Arctic Front Advance, Medtronic) and circular catheter (Achieve 20 mm, Medtronic) were placed in the LA. Attempts were made to record the pulmonary venous potential (PVP) in each PV. We performed a TTI-based ablation protocol. The dosing of CBA was as follows:

1. If the PVP was recorded and the TTI was less than 60 s, the duration of CBA was between TTI + 90 s and TTI + 120 s at the operator's discretion, and a bonus CBA of 120 s was applied.
2. If the TTI was between 60 s and 90 s, the duration of CBA was 180 s and a bonus CBA of 120 s was applied.
3. If PVI was not achieved within 90 s, CBA was abandoned and the balloon was repositioned for a subsequent CBA.
4. If the PVP was not recorded during CBA, an empiric CBA was delivered for 120 s. If PVI was achieved after this CBA, a bonus of between 160 s and 180 s was applied; if not, the balloon was repositioned for a subsequent CBA.

During the bonus application (point 4), the operators were encouraged to change the balloon's position and orientation by placing the Achieve catheter in a different PV branch. If the nadir temperature of the balloon was equal to or less than −55°C and the duration of ablation was less than 120 s, the CBA was stopped in advance and the balloon repositioned for a subsequent CBA. If the nadir temperature was equal to or less than −55°C and the duration of ablation was already above 120 s, CBA was ceased in advance and no additional CBA was given. During the CBA of right-sided PVs, phrenic movement was monitored by continuous phrenic nerve stimulation with a catheter positioned in the superior vena cava. Complete PVI was considered a bidirectional conduction block between LA and PV. Cardioversion was applied when the heart rhythm was still AF following completion of CBA.

### Data collection

The data were collected for analysis including: (1) Basic demographics; (2) Imaging result of LA diameter was derived from echocardiography by measuring the anteroposterior diameter of LA from the parasternal transverse axis. Left ventricular ejection fraction was measured by Simpson's biplane method. LA Volume (LAV) was derived from CTA. The original scanned images were processed by the in-built software of the CT for the construction of the three-dimension model of the LA and PVs. PVs were removed from the three-dimension model and the LAV was calculated automatically. (3) peri-procedural characteristics such as recording of PVP, TTI, balloon temperature during cryoablation.

### Follow-up

All patients were hospitalized with rhythm monitored by telemetry for 3 continuous days after CBA. Patients were followed up with 24-h Holter electrocardiogram every 3 months over the first year and every 6 months thereafter. OAC was continued for at least 8 weeks and prescription of AADs was allowed during the blanking period at the discretion of the clinical cardiologist. AF recurrence was defined as atrial arrhythmia persisting more than 30 s—including AF, atrial flutter, or atrial tachycardia—beyond a 90-day blanking period (the period during which recurrence is considered clinically insignificant). In patients with symptoms suggestive of PV stenosis or in those undergoing a CBA procedure, CT of the LA and PV was performed to exclude PV stenosis.

### Statistical analysis

Statistical analyses were performed using SPSS 24.0 (IBM Inc., Chicago, IL, USA), X-tile 3.6.1, and R (version 4.0.2; R Foundation for Statistical Computing, Vienna, Austria) software. Continuous data are presented as means with standard deviation, and categorical variables are given as numbers and percentages. Student's *t*-test and the chi-square test were used to compare clinical characteristics and variables as appropriate. Survival free from atrial arrhythmia was estimated by the Kaplan-Meier method and compared by log-rank tests. Cox proportional hazards models were used to derive hazard ratios and the corresponding confidence intervals.

All 498 patients were randomly divided into a training cohort and a validation cohort (7:3) based on complete data. The training cohort was used to develop the model, and the validation cohort was applied to validate the model. We used a forward + backward stepwise elimination approach to identify predictive variables for the model. Least absolute shrinkage and selection operator regression was also applied in the predictor's selection to examine the importance of predictive variables selected by stepwise regression analysis. Based on the selected predictive variables, the Cox regression model was developed and presented as the nomogram. We assessed the predictive accuracy of the nomogram after discrimination and calibration. To quantify the discrimination performance of the nomogram, Harrell's C-index was measured. Calibration curves, accompanied by the Hosmer-Lemeshow test, were plotted to assess the nomogram's calibration. To assess the nomogram's performance, the Cox regression formula developed in the training cohort was then applied in the validation cohort and predicted survival was calculated.

We analyzed the following risk scores specifically developed for the prediction of AF recurrence post-CBA: CHA_2_DS_2_-VASc, SCALE-CryoAF, MB-LATER, CAAP-AF, and BASE-AF_2_. DeLong's test was used to compare the C-index of the nomogram and the scores in the training and validation cohorts. Time-dependent receiver operating characteristic (ROC) curve was evaluated and the area under curve (AUC) was calculated for the discriminative power of the nomogram and the scores. In addition, we performed a decision curve analysis of the monogram and the two scores.

All *P* values were two-sided, with *P* < . 05 indicating statistical significance. C-index, calibration curve, nomogram, and bootstrapping validation were calculated or formulated using the rms and risk regression packages in R.

## Results

### Patients' baseline demographics

As shown in Table 1, the mean age of our 498 patients was 59.9 years; 63.9% were male, 36.1% were female, and 29.5% of the total had persistent AF. We analyzed the clinical baseline characteristics of patients with and without recurrence and found that there was no difference in terms of age, gender, AF diagnosis time, or AF-related comorbidities. Compared with patients without recurrence, patients with recurrence had a higher percentage of persistent AF, larger LA, and higher clinical score.

**Table 1:** Baseline clinical characteristics.

	All patients (*n* = 498)	No recurrences (*n* = 321)	Recurrences (*n*= 177)	*P* value
Age (year)	59.9 ± 10.3	59.8 ± 10.5	60.1 ± 10.0	0.769
Sex (Male)	63.9%	66.7%	58.8%	0.079
Body mass index (kg/m2)	24.6 ± 3.0	24.6 ± 3.1	24.7 ± 2.8	0.594
Months since first AF diagnosis	37.9 ± 36.0	35.7 ± 34.7	41.7 ± 38.1	0.076
Persistent AF	29.5%	22.7%	41.8%	<0.001
Hypertension	54.6%	52.6%	58.2%	0.234
Diabetes mellitus	10.6%	11.8%	8.5%	0.244
Coronary artery disease	10.6%	11.2%	9.6%	0.577
Heart failure	0.6%	0.6%	0.6%	0.936
Previous Stroke/TIA	1.2%	1.6%	0.6%	0.331
Left atrial diameter	40.2 ± 4.0	39.8 ± 3.7	41.1 ± 4.5	<0.001
LVEF	65.9 ± 5.3	66.0 ± 5.3	65.6 ± 5.2	0.407
Left atrial volume	115.4 ± 38.9	110.4 ± 31.9	124.3 ± 47.9	<0.001
CHA_2_DS_2_VASc score	1.7 ± 1.4	1.7 ± 1.4	1.7 ± 1.3	0.781
BASE-AF2	1.3 ± 1.1	1.1 ± 1.0	1.7 ± 1.0	<0.001
SCALE-CryoAF	2.1 ± 2.4	1.5 ± 1.9	3.3 ± 2.6	<0.001
CAAP-AF	3.9 ± 1.5	3.7 ± 1.5	4.2 ± 1.5	<0.001
MBLATER	1.2 ± 0.9	0.9 ± 0.7	1.5 ± 1.0	<0.001

AF, Atrial fibrillation; TIA, transient ischemic attack; LVEF, left ventricular ejection fraction.

### Stratification of patients according to procedural and biophysical characteristics

Compared with patients without long-term recurrence, patients with long-term recurrence had longer procedural and LA dwell times as well as higher fluoroscopic doses. In patients without recurrence, PVP was more frequently recorded; average TTI was shorter, and there were fewer unsuccessful freezes. Regarding each PV, it was noticed that the balloon temperature at 30 s during successful CBA (Temp_30_), the balloon temperature at 60 s (Temp_60_), and the nadir balloon temperature (Temp_nadir_) were significantly lower and TTI was significantly shorter in patients without recurrence. Details of the biophysical characteristics are shown in Table 2.

**Table 2:** Procedure-related biophysical characteristics and data.

	All patients (*n* = 498)	No recurrences (*n* = 321)	Recurrences (*n* = 177)	*P* value
Procedure duration (min)	85.3 ± 33.2	81.2 ± 29.2	92.6 ± 38.4	0.001
LA dwell time (min)	61.0 ± 29.7	58.2 ± 26.8	66.1 ± 33.8	0.005
Fluoroscopy time (min)	13.3 ± 6.0	12.3 ± 5.3	15.1 ± 6.7	<0.001
Radiation dose (mGray)	286.4 ± 192.1	270.4 ± 185.1	315.3 ± 201.4	0.014
Number of PV with real-time recording of PV electrogram	2.5 ± 1.2	2.7 ± 1.1	2.1 ± 1.2	<0.001
Average TTI	38.4 ± 10.4	36.7 ± 9.6	41.7 ± 11.1	<0.001
Total number of freezes	10.0 ± 2.7	9.9 ± 2.5	10.2 ± 3.0	0.283
Total number of unsuccessful freezes	2.4 ± 2.4	2.1 ± 2.4	2.8 ± 2.5	0.007
LSPV				
Total number of freezes	2.9 ± 1.4	2.7 ± 1.3	3.2 ± 1.6	<0.001
Number of unsuccessful freezes	0.9 ± 1.3	0.7 ± 1.2	1.2 ± 1.5	<0.001
Total duration of freezes	399 ± 163.2	379.2 ± 145.4	435.3 ± 186.7	<0.001
Average duration of freezes	142.7 ± 18.5	144.9 ± 18.3	138.8 ± 18.2	0.001
Temp_30_ (°C)	−29.9 ± 4.4	−30.3 ± 4.2	−29.3 ± 4.6	0.010
Temp_60_ (°C)	−40.4 ± 4.8	−40.9 ± 4.5	−39.4 ± 5.1	0.001
Temp_nadir_ (°C)	−47.1 ± 5.0	−47.5 ± 4.9	−46.4 ± 5.0	0.020
Real-time recording of PV electrogram	78.7%	84.7%	67.8%	<0.001
TTI	42.5 ± 16	41.7 ± 16.1	44.5 ± 15.7	0.773
LIPV				
Total number of freezes	2.3 ± 0.9	2.3 ± 0.9	2.1 ± 0.8	0.005
Number of unsuccessful freezes	0.4 ± 0.7	0.4 ± 0.8	0.3 ± 0.6	0.256
Total duration of freezes	339.1 ± 118	351.4 ± 113.1	316.6 ± 123.6	0.002
Average duration of freezes	152.2 ± 18.1	153.0 ± 17.3	150.7 ± 19.5	0.180
Temp_30_ (°C)	−28.7 ± 3.9	−29.3 ± 3.7	−27.5 ± 3.8	<0.001
Temp_60_ (°C)	−37.7 ± 4.2	−38.5 ± 4.0	−36.2 ± 4.2	<0.001
Temp_nadir_ (°C)	−43.2 ± 4.7	−43.9 ± 4.5	−41.8 ± 4.7	<0.001
Real-time recording of PV electrogram	66.3%	72.3%	53.4%	0.001
TTI	35.8 ± 15.3	33.5 ± 14.6	41.3 ± 15.6	<0.001
RSPV				
Total number of freezes	2.5 ± 1.0	2.5 ± 1.0	2.5 ± 1.1	0.963
Number of unsuccessful freezes	0.5 ± 1.0	0.5 ± 1.0	0.6 ± 0.9	0.294
Total duration of freezes	327.1 ± 124.1	327.4 ± 121.5	326.5 ± 129.2	0.937
Average duration of freezes	136.6 ± 19.8	136.3 ± 18.9	137.2 ± 21.4	0.625
Temp_30_ (°C)	−31.8 ± 4.6	−32.6 ± 4.5	−30.2 ± 4.5	<0.001
Temp_60_ (°C)	−42.2 ± 5.2	−43.2 ± 4.9	−40.3 ± 5.3	<0.001
Temp_nadir_ (°C)	−49.5 ± 5.7	−50.7 ± 5.0	−47.2 ± 6.3	<0.001
Real-time recording of PV electrogram	61.7%	69.8%	46.9%	<0.001
TTI	33.8 ± 15	32.0 ± 13.8	38.7 ± 16.8	<0.001
RIPV				
Total number of freezes	2.4 ± 1.2	2.4 ± 1.1	2.4 ± 1.3	0.925
Number of unsuccessful freezes	0.5 ± 1.1	0.5 ± 1.0	0.6 ± 1.1	0.246
Total duration of freezes	333.6 ± 144.1	332.9 ± 131.6	334.8 ± 164.8	0.891
Average duration of freezes	143.0 ± 22.1	143.0 ± 22.7	142.9 ± 20.9	0.977
Temp_30_ (°C)	−30.2 ± 4.9	−31.0 ± 4.9	−28.9 ± 4.6	<0.001
Temp_60_ (°C)	−39.8 ± 5.2	−40.7 ± 5.2	−38.3 ± 4.9	<0.001
Temp_nadir_ (°C)	−46.3 ± 6.3	−47.3 ± 6.2	−44.5 ± 6.1	<0.001
Real-time recording of PV electrogram	52.8%	59.2%	41.2%	<0.001
TTI	38.9 ± 15.1	37.7 ± 15.4	42.1 ± 13.9	0.032

LSPV, left superior pulmonary vein; LIPV, left inferior pulmonary vein; RSPV, right superior pulmonary vein; RIPV, right inferior pulmonary vein; TTI, time to pulmonary vein isolation; Temp_30_, Temperature of CB at 30 s; Temp_60_, Temperature of CB at 60 s; Temp_nadir_, nadir temperature of CB.

Stratification of the balloon temperature and TTI of each PV was performed by X-tile and the optimal cutoff values were determined (Table 3). Scores corresponding to Temp_30_, Temp_60_, Temp_nadir_, and TTI were calculated by counting the number of PVs achieving the cutoff value; these ranged from 0 to 4. Number of PVs with real-time recording of PVP and number of unsuccessful freezes were counted and analyzed. The results are shown in Table 4.

**Table 3:** Cutoff value of balloon temperature and TTI of each PV.

	Cutoff value	*X* ^2^	*P* value
LSPV			
TTI	48 s	9.872	0.039
Temp_30_	−26°C	6.957	0.138
Temp_60_	−36°C	14.470	0.005
Temp_nadir_	−47°C	9.872	0.039
LIPV			
TTI	40 s	30.517	<0.001
Temp_30_	−27°C	38.682	<0.001
Temp_60_	−36°C	38.063	<0.001
Temp_nadir_	−40°C	32.291	<0.001
RSPV			
TTI	40 s	17.379	0.001
Temp_30_	−31°C	23.333	<0.001
Temp_60_	−42°C	33.126	<0.001
Temp_nadir_	−47°C	50.187	<0.001
RIPV			
TTI	39 s	12.664	0.011
Temp_30_	−28°C	19.176	0.001
Temp_60_	−37°C	24.695	<0.001
Temp_nadir_	−43°C	25.483	<0.001

**Table 4:** Distribution of patients with number of PVs with real-time recording of PV potential, TTI score, balloon temperature scores and number of unsuccessful freezes.

	All patients (*n* = 498)	No Recurrences (*n* = 321)	Recurrences (*n* = 177)	*P* value
Number of PVs with real-time recording of PV potential				
0	21 (4.2%)	4 (1.2%)	17 (9.6%)	<0.001
1	69 (13.9%)	31 (9.7%)	38 (21.5%)	
2	131 (26.3%)	73 (22.7%)	58 (32.8%)	
3	147 (29.5%)	111 (34.6%)	36 (20.3%)	
4	130 (26.1%)	102 (31.8%)	28 (15.8%)	
TTI score				
0	80 (16.1%)	16 (5%)	64 (36.2%)	<0.001
1	143 (28.7%)	82 (25.5%)	61 (34.5%)	
2	129 (25.9%)	99 (30.8%)	30 (16.9%)	
3	103 (20.7%)	85 (26.5%)	18 (10.2%)	
4	43 (8.6%)	39 (12.1%)	4 (2.3%)	
Score of Temp_30_				
0	28 (5.6%)	8 (2.5%)	20 (11.3%)	<0.001
1	66 (13.3%)	31 (9.7%)	35 (19.8%)	
2	104 (20.9%)	57 (17.8%)	47 (26.6%)	
3	151 (30.3%)	105 (32.7%)	46 (26%)	
4	149 (29.9%)	120 (37.4%)	29 (16.4%)	
Score of Temp_60_				
0	23 (4.6%)	4 (1.2%)	19 (10.7%)	<0.001
1	69 (13.9%)	30 (9.3%)	39 (22%)	
2	111 (22.3%)	59 (18.4%)	52 (29.4%)	
3	149 (29.9%)	110 (34.3%)	39 (22%)	
4	146 (29.3%)	118 (36.8%)	28 (15.8%)	
Score of Temp_nadir_				
0	36 (7.2%)	9 (2.8%)	27 (15.3%)	<0.001
1	59 (11.8%)	24 (7.5%)	35 (19.8%)	
2	108 (21.7%)	72 (22.4%)	36 (20.3%)	
3	176 (35.3%)	120 (37.4%)	56 (31.6%)	
4	119 (23.9%)	96 (29.9%)	23 (13%)	
Number of unsuccessful freezes				
0	122 (24.5%)	89 (27.7%)	33 (18.6%)	<0.001
1	105 (21.1%)	75 (23.4%)	30 (16.9%)	
2	90 (18.1%)	61 (19%)	29 (16.4%)	
≥3	181 (36.3%)	96 (29.9%)	85 (48%)	

### Long-term outcome after cryoballoon ablation and risk stratification by score

**Figure 1:**
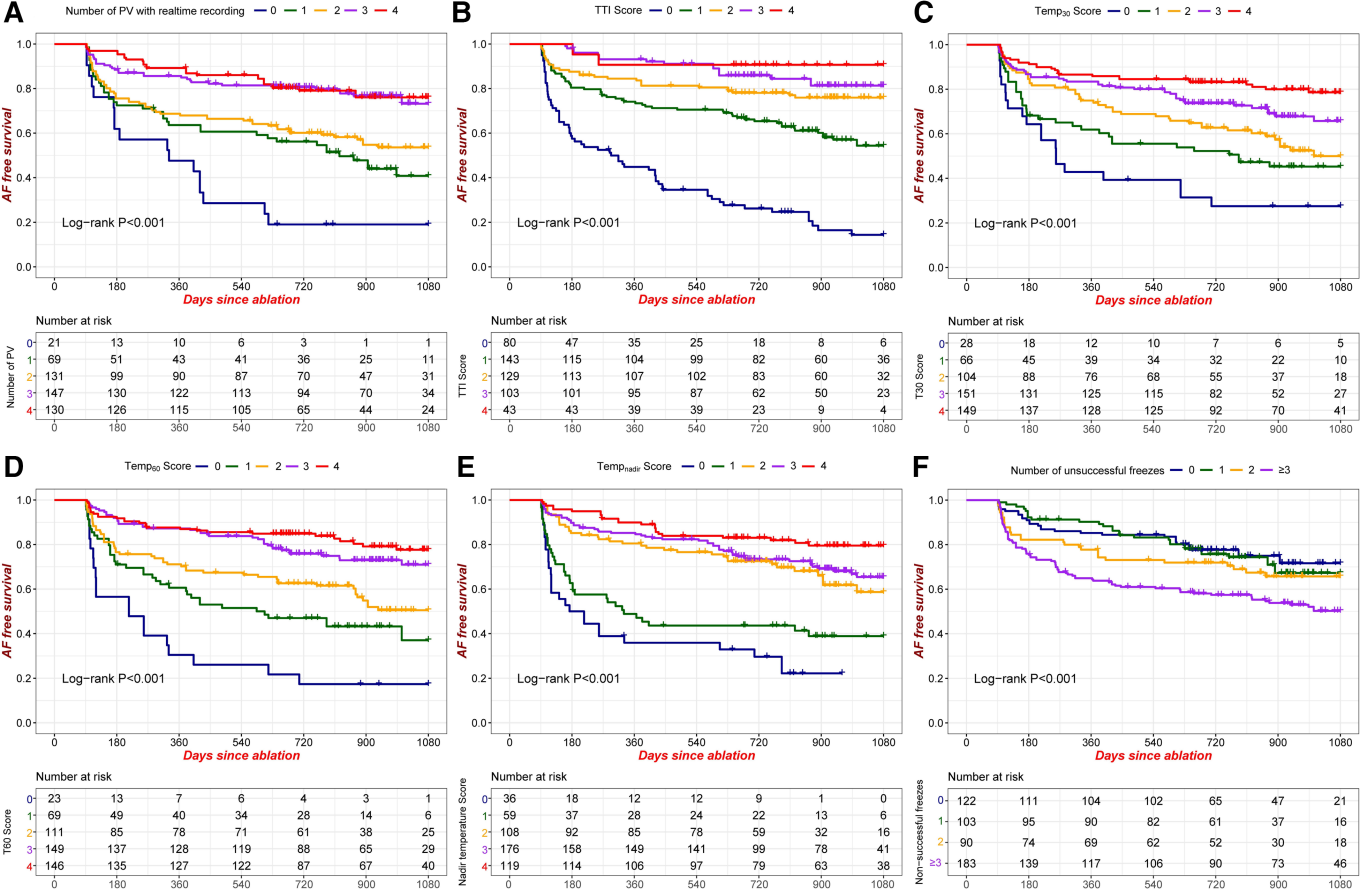
Kaplan-Meier AF-free survival according to different criteria. (**A**) Number of PVs with real-time recording of PVP; (**B**) TTI score; (**C**) Temp_30_ score; (**D**) Temp_60_ score; (**E**) Temp_nadir_ score; (**F**) Number of unsuccessful freezes. PVP: pulmonary venous potential; TTI score: number of PVs achieving the cutoff value of TTI; Temp_30_ score: number of PVs achieving the cutoff value of balloon temperature at 30 s; Temp_60_ score: number of PVs achieving the cutoff value of balloon temperature at 60 s; Temp_nadir_ score: number of PVs achieving the cutoff value of nadir balloon temperature.

AF recurrence was seen in 177 of the total number of 498 patients. The Kaplan–Meier estimated AF-free survival was 77.4% at 1 year, 68.7% at 2 years, and 60.5% at 3 years. Kaplan–Meier AF-free survival curves with regard to the TTI score, the balloon temperature score (Temp_30_, Temp_60_, Temp_nadir_), the PV number with real-time recording of PVP, and the number of unsuccessful freezes are presented in Figure 1. These variables showed predictive ability for recurrence. Thus, our results support the idea that these factors can be useful for risk stratification and for the prediction of outcome after CBA for AF.

### Factor selection and nomogram construction

We randomly allocated 69% (342) of our patients to the training cohort and the remaining 31% (156) to the validation cohort. There were 123 (36.0%) patients in the training cohort and 52 (33.3%) in the validation cohort who experienced recurrence after CBA. There were no significant differences between the training and validation cohorts regarding preoperative baseline and ablation characteristics (Supplementary Table S1).

**Figure 2:**
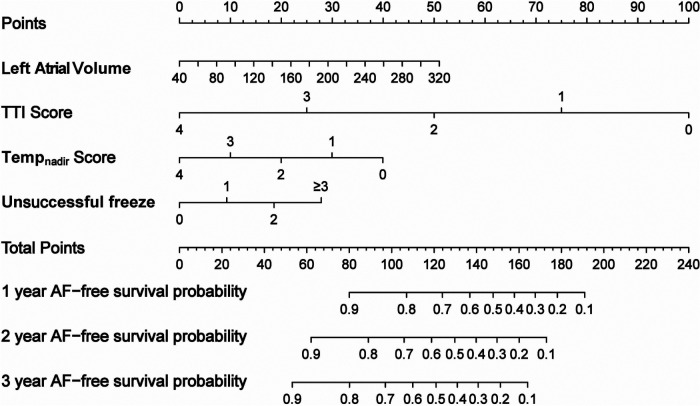
Nomogram derived from the training cohort for the prediction of AF recurrence after cryoballoon ablation.

Stepwise regression analysis and multivariate Cox regression revealed that the LAV, TTI score, Temp_nadir_ score, and number of unsuccessful freezes were identified as significant independent risk factors for AF recurrence (Supplementary Table S2 and Table 5). The nomogram based on these four factors from the training cohort was developed for the prediction of 1-, 2-, and 3-year AF-free survival. The total score, obtained by adding the scores for each of the four factors, was predictive of the 1-, 2-, and 3-year AF-free survival for each individual patient in the training cohort (Figure 2).

**Table 5:** Cox regression analysis results of recurrence risk factors of atrial fibrillation patients after CB ablation.

Factor	HR	95%CI	*P* value
LAV	1.006	1.002–1.010	0.006
TTI Score	0.495	0.404–0.605	<0.001
Temp_nadir_ Score	0.759	0.649–0.888	<0.001
Number of unsuccessful freezes	1.298	1.108–1.519	0.001

HR, hazard ration; CI, confidence interval.

### Validation and calibration of the nomogram

**Figure 3:**
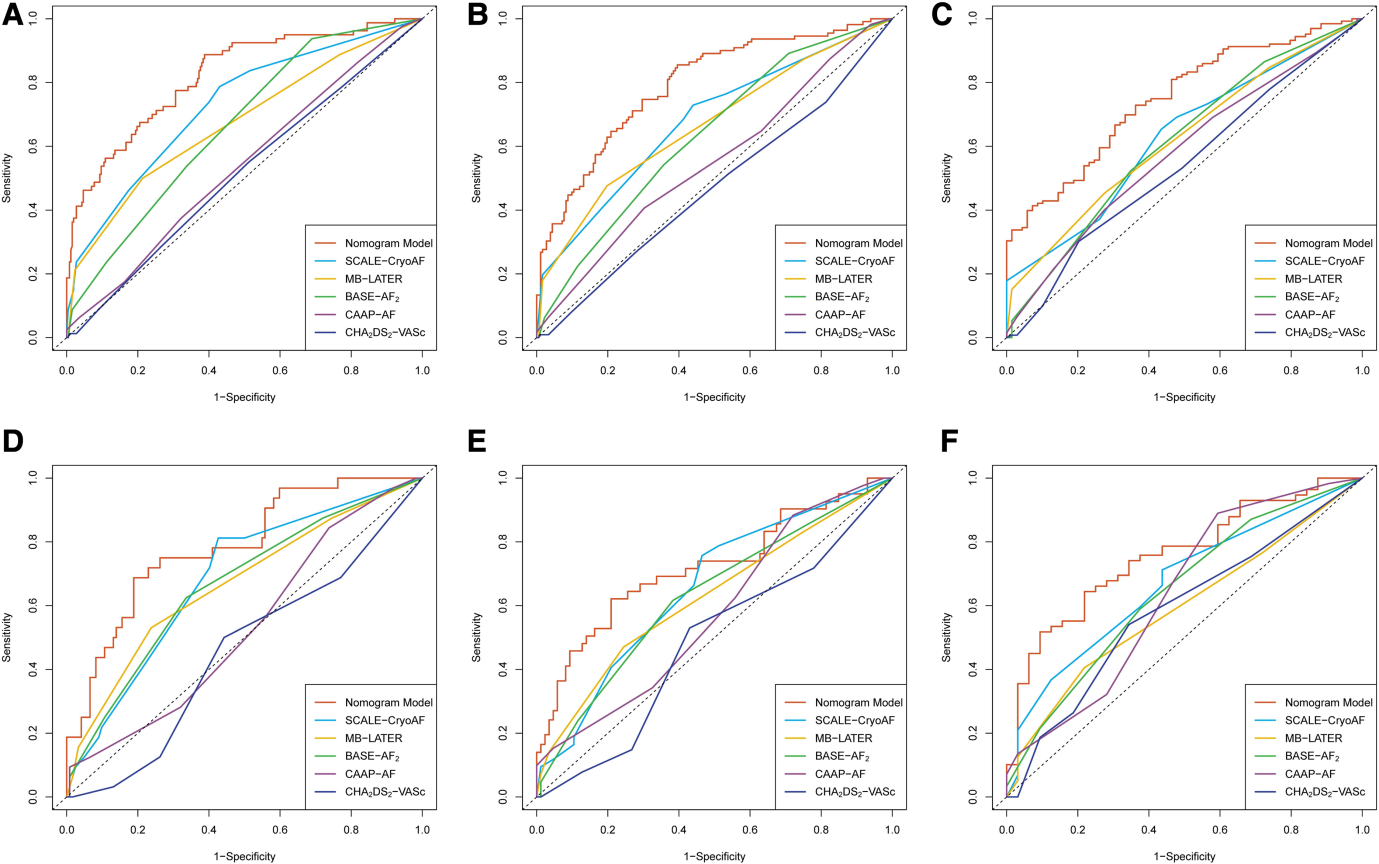
Receiver operating characteristic curves of the nomogram model and the conventional risk scores in the training cohort for (**A**) the prediction of 1-year recurrence; (**B**) prediction of 2-year recurrence; and (**C**) the prediction of 3-year recurrence. In the validation cohort, (**D**) the prediction of 1-year recurrence; (**E**) the prediction of 2-year recurrence; and (**F**) the prediction of 3-year recurrence.

**Figure 4:**
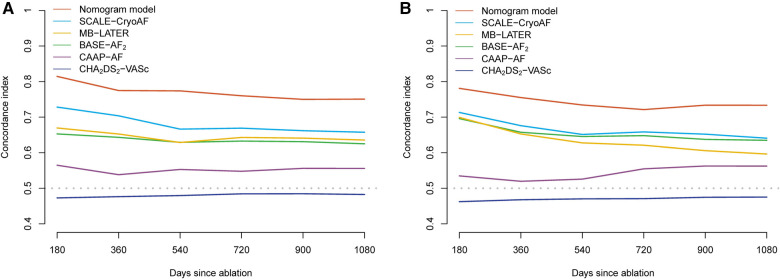
C-index of the nomogram model and conventional risk scores in training cohort (**A**) and the validation cohort (**B**).

ROC curves were used to evaluate the nomogram's predictive ability for 1-, 2-, and 3-year AF-free survival in both the training and validation cohorts. Our nomogram demonstrated good discriminative ability in both the training cohort (1-year AUC, 82.1%; 2-year AUC, 79.3%; 3-year AUC, 76.2%) and the validation cohort (1-year AUC, 78.6%; 2-year AUC, 71.9%, 3-year AUC, 75.7%) for AF-free survival rates (Figure 3). In comparison with other prediction models based on clinical characteristics, our nomogram demonstrated better accuracy with significance in predicting recurrence in the validation cohort. Although statistical differences between the nomogram models and conventional risk scores were not significant, it was noticed that the AUCs were greater for our nomogram model (Table 6). In addition, the C-index of the nomogram model was greater than the C-index of the conventional risk scores in both the training and validation cohorts (Figure 4).

**Table 6:** Results of AUCs of time dependent ROC curves in training and validation cohorts.

	1 Year		2 Year		3 Year	
	AUC% (95%CI)	*P*	AUC% (95%CI)	*P*	AUC% (95%CI)	*P*
Training set						
Nomogram	82.1 (76.7–87.5)	–	79.3 (73.9–84.6)	–	74.8 (67.9–81.7)	–
SCALE-CryoAF	72.8 (66.6–79.0)	0.017	67.8 (61.6–74.0)	0.003	62.9 (55.0–70.7)	0.012
MB-LATER	67.3 (60.7–74.0)	<0.001	66.7 (60.6–72.8)	0.001	62.2 (54.6–69.9)	0.012
BASE-AF_2_	66.4 (60.3–72.5)	<0.001	63.4 (57.2–69.6)	<0.001	61.3 (53.2–69.4)	0.009
CAAP-AF	53.9 (46.9–60.9)	<0.001	55.2 (48.5–61.9)	<0.001	57.9 (49.9–66.0)	0.001
CHA_2_DS_2_-VASc	51.6 (44.6–58.7)	<0.001	47.0 (40.3–53.7)	<0.001	53.9 (45.5–62.2)	<0.001
Validation set						
Nomogram	78.6 (69.9–87.3)	–	71.9 (61.8–81.9)	–	75.7 (65.4–86.0)	–
SCALE-CryoAF	68.5 (58.9–78.2)	0.110	65.6 (56.1–75.3)	0.345	66.9 (55.7–78.2)	0.018
MB-LATER	66.8 (56.4–77.2)	0.057	62.5 (52.4–72.7)	0.167	59.2 (47.4–70.9)	0.630
BASE-AF_2_	66.6 (56.3–76.8)	0.034	62.9 (52.9–72.9)	0.173	64.5 (52.8–76.3)	0.111
CAAP-AF	52.9 (42.3–63.6)	<0.001	57.5 (47.2–67.7)	0.035	63.7 (51.3–76.2)	0.098
CHA_2_DS_2_-VASc	45.7 (35.1–56.3)	<0.001	48.3 (37.9–58.7)	0.001	58.6 (46.2–71.1)	0.040

AUC, area under curve; ROC, receiver operating characteristic; CI, confidence interval.

**Figure 5:**
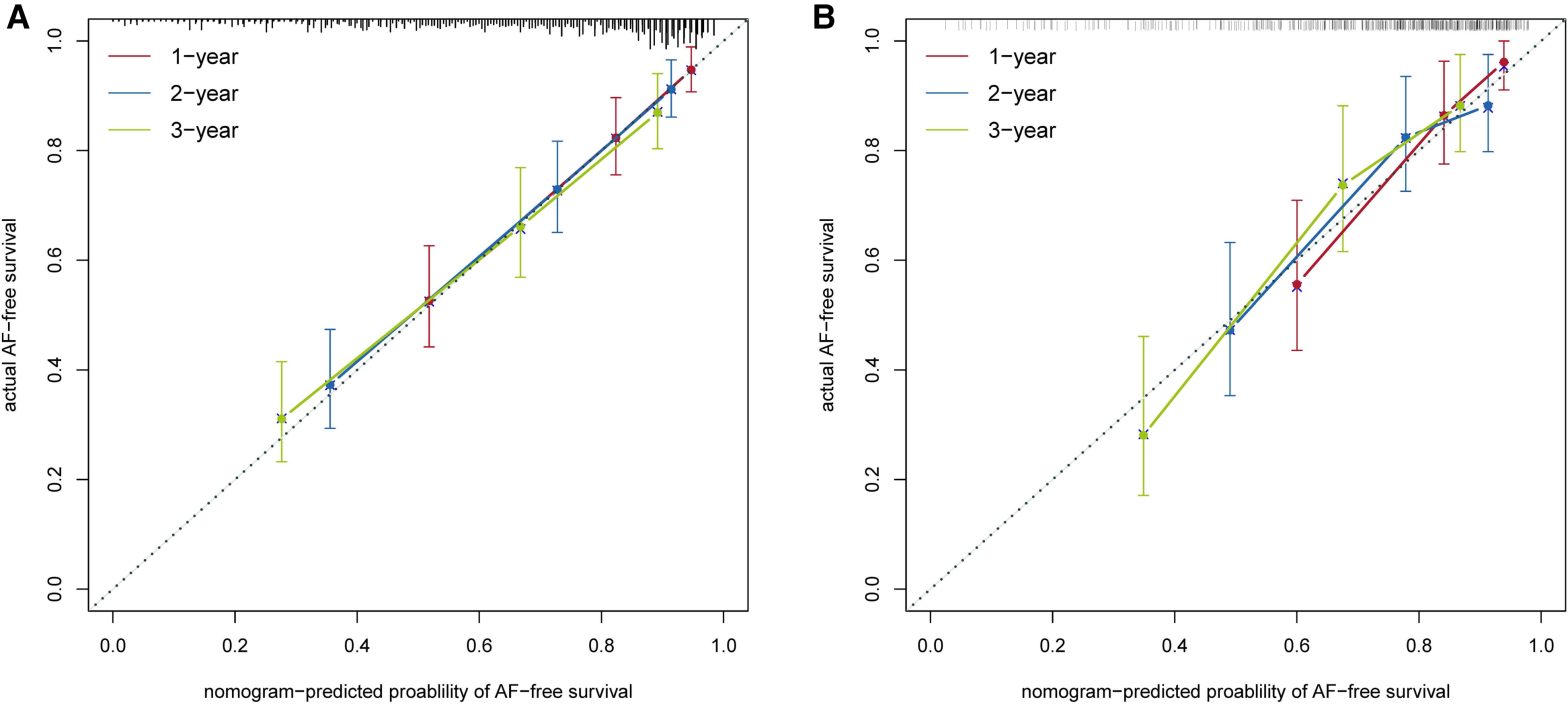
Calibration curves of the nomogram model in the training cohort (**A**) and in the validation cohort (**B**).

The nomogram had acceptable calibration in the training cohort (Hosmer-Lemeshow statistics: 1 year, *χ*^2 ^= 10.278, *P* = . 246; 2 years, *χ*^2 ^= 5.209, *P* = . 735; 3 years, *χ*^2 ^= 3.924, *P* = . 864) and the validation cohort (Hosmer-Lemeshow statistic: 1 year, *χ*^2 ^= 14.552, *P* = . 068; 2 years, *χ*^2 ^= 6.378, *P* = . 605; 3 years, *χ*^2 ^= 7.889, *P* = . 444). The calibration plots of our nomogram also showed optimal agreement between the actual observations and the predicted outcomes both in the training cohort and the validation cohort (Figure 5) for all time points. Thus these nomogram-based results display good accuracy for predicting 1-, 2-, and 3-year AF-free survival after CBA.

**Figure 6:**
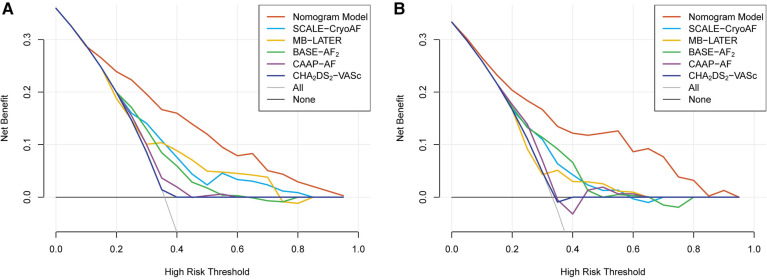
The results of decision curves analysis (DCA) of the nomogram model and the conventional risk scores in the training cohort (**A**) and in the validation cohort (**B**).

Compared with the other prediction models, the results of the decision curve analyses (DCAs) demonstrate that the nomogram model has good clinical effectiveness in both the training and validation cohorts (Figure 6). All the results indicate that the accuracy, discriminative ability, and clinical effectiveness of the nomogram model are superior to those of the other conventional risk scores.

## Discussion

To our knowledge, this is the first nomogram developed for the prediction of AF recurrence using both clinical characteristics and procedural biophysical parameters during CBA. The nomogram performed well in both the training and validation cohorts. The model contains only four variables (LAV, TTI score, Temp_nadir_ score, and number of unsuccessful freezes), all of which are available and easy to use in clinical practice. In comparison with other conventional risk scores, our nomogram showed better predictive ability and good potential implication in clinical practice.

### Factors related to recurrence after cryoballoon ablation

We discovered that LAV, TTI, Temp_nadir_, and number of unsuccessful freezes were associated with AF recurrence. It is certain that LAV was highly associated with the success of CBA. Patients with an enlarged LA—which contains more extra-PV triggers and arrhythmogenic substrate than a normal LA (owing to electrical remodeling, structural remodeling, and interstitial fibrosis)—are at greater risk for the recurrence of atrial arrythmia after an initial CBA (5, 6). The type of AF was unexpectedly not included in the related factors, for which we speculate that LAV is quantitative and more representative of persistency of AF and severity of LA remodeling, which is greatly associated with the efficacy outcome of cryoballoon ablation. However, the number of unsuccessful freezes also reflects the development of AF from a different angle. The increased number of unsuccessful freezes may derive from anatomic variation, dilation of the PV ostium, and enlargement of the LA, which have already been proven to raise the risk of AF recurrence after CBA (7, 8).

It is well established that PVI is the cornerstone of AF ablation. Since CBA was designed for the convenience of PVI, the procedural biophysical parameter is considered an important indicator of sufficient ablation. Several studies have already reported that a longer TTI is associated with early reconnection of the PV and a higher risk of recurrence after CBA (4, 9, 10). Therefore, TTI has emerged as an important marker for the dosing of CBA. Several studies that have designed a CBA dosing protocol based on TTI achieved a noninferior efficacy outcome compared with conventional protocols or radiofrequency ablation (11–13). However, the cutoff value of TTI was the same for the four PVs in those studies despite the different anatomic characteristics of each PV. As shown in Table 2, the average values of TTI differed among the four PVs. In our study, therefore, we analyzed the four PVs separately and explored the best cutoff value for each. By calculating and aggregating the score of each PV, the TTI score better reflected the durability of PVI and is believed to be more accurate and reliable for the prediction of AF recurrence.

Balloon temperature during CBA affects the occlusion of the treated PV. Lower balloon temperature reflects better balloon-tissue contact. Thus, the balloon temperature is known to be an important indicator of CBA efficiency. Fürnkranz reported that the nadir temperature is predictive of the acute outcome of PVI and helpful in identifying early PV reconnection (14). In addition, several studies have identified the role of balloon temperature in predicting long-term durable PVI (15, 16). The optimal balloon temperature during CBA remains unclear, but there a prospective study using CBA guided by balloon temperature has already demonstrated that cryoapplication with a balloon temperature lower than −30°C within 40 s showed good acute outcomes of PVI and comparable clinical efficacy and safety profiles (17). Accordingly, it is well recognized that balloon temperature is an important indicator of durable PVI as well as procedural success. In addition, the optimal criteria for nadir temperature of each PV differed among the four PVs (18). Therefore we determined the optimal cutoff of TTI and balloon temperature for the four PVs. This is believed to represent the biophysical characteristics of CBA efficiency. In our nomogram model, we included the balloon temperature and calculated the Temp_nadir_ score. The result of our study also emphasizes the importance of monitoring balloon temperature during CBA.

### Advantages of the nomogram model

The monogram model was superior to the general score (CHA_2_DS_2_-VASc) and specific scores (SCALE-CryoAF, MB-LATER, CAAP-AF, and BASE-AF_2_) in predicting and discriminating recurrence. These scores were basically calculated using clinical factors such as age, type of AF, duration of AF in the past, history of coronary heart disease, left ventricular ejection fraction, LA diameter, and other factors. However, the biophysical parameters of CBA were not included in those scores, although it has been reported that these biophysical parameters are associated with acute outcome of PVI (4). Our study shows that the C-index of our nomogram model was larger in either the training cohort or the validation cohort than that of other prediction models. In addition, this supported the fact that high quality of PVI was equally crucial for the outcome as clinical factors. In recently published studies, radiofrequency ablation using AI technology yield higher PVI durability and better efficacy outcome (19, 20). Although those studies were performed in radiofrequency ablation, it is undoubtable that durable PVI after CBA was similarly associated with a favorable efficacy outcome.

Furthermore, our monogram model balanced the contribution of each factor to CBA outcome. In conventional risk scores, the coefficient of each risk factor usually comprised an integer. In our nomogram model, however, the coefficients were calculated based on the significance of the risk factor. Therefore, it is reasonable that the monogram model had an advantage over conventional risk scores. In addition, our model uses only four factors, which are available and easy to calculate during procedure. In specific patients with high scores, the electrophysiologist performing CBA should pay more attention to non-PV triggers or the arrhythmogenic substrate and acute PV reconnection. It may also be necessary to extend the ablation range and verify PVI durability. In summary, our nomogram model combines both clinical factors and biophysical parameters, which raises its predictive performance compared with conventional risk scores and implies its clinical significance for the guidance of CBA.

## Limitations

Our study has several limitations. First, this was a retrospective analysis in which selection bias may have existed; therefore, a future prospective study is warranted. Second, the patients' data were obtained from our center only, and no external validation was applied. Although we have separated the total patients into two cohorts, external validation of our results was still required before our findings could be applied clinically. Finally, the variables included in our study were based on our routine clinical practice. Regarding biophysical parameters, we collected only real-time recordings of PVP, CB temperature during freezes, TTI, and number of unsuccessful freezes. Other parameters such CB warming time were not included. The inclusion of more parameters might further improve the accuracy of the model.

## Conclusions

This study presents a nomogram that is easy to apply and can predict the long-term efficacy and outcome of CBA. Biophysical parameters such as TTI and cryoballoon temperature have a great impact on AF recurrence. The nomogram has been shown to be superior in its predictive accuracy as compared with assessments based on conventional risk scores.

## Data Availability

The raw data supporting the conclusions of this article will be made available by the authors, without undue reservation.
