# Supplementary material for: Development and validation of a novel nomogram for predicting recurrent atrial fibrillation after cryoballoon ablation
Source: Front Cardiovasc Med. 2023 Aug 11;10:1073108. doi: 10.3389/fcvm.2023.1073108 (PMC10453796; doi:10.3389/fcvm.2023.1073108)
Supplement: Supplementary file 1 [file Table_1.docx]

Supplementary Materials Legends

Supplementary table 1: Comparison of clinical, procedural and biophysical characteristics of training and validation cohorts

Supplementary table 2: Result of the stepwise regression analysis in a forward approach for variable selection

Supplementary table 1: Comparison of clinical, procedural and biophysical characteristics of training and validation cohorts

|  | Training cohort  (n=344) | Validation cohort  (n=154) | P value |
| --- | --- | --- | --- |
| Age (year) | 60.2±10.4 | 59.4±10.2 | 0.430 |
| Sex (Male) | 62.5% | 66.9% | 0.348 |
| Body mass index (kg/m2) | 24.6±2.9 | 24.8±3.1 | 0.513 |
| Months since first AF diagnosis | 37.4±36.1 | 38.8±36.1 | 0.690 |
| Persistent AF | 28.8% | 31.2% | 0.590 |
| Hypertension | 54.9% | 53.9% | 0.829 |
| Diabetes mellitus | 9.9% | 12.3% | 0.413 |
| Coronary artery disease | 9.3% | 13.6% | 0.148 |
| Heart failure | 0.9% | 0% | 0.246 |
| Previous Stroke/TIA | 1.7% | 0% | 0.100 |
| Left atrial diameter(mm) | 40.4±4.1 | 39.9±3.9 | 0.207 |
| LVEF(%) | 65.8±5.5 | 66.1±5.0 | 0.554 |
| Left atrial volume(ml) | 117.1±39.6 | 111.5±37 | 0.137 |
| CHA_2_DS_2_VASc score | 1.7±1.4 | 1.7±1.3 | 0.905 |
| BASE-AF_2_ | 1.3±1 | 1.3±1.1 | 0.888 |
| SCALE-CryoAF | 2.1±2.4 | 2.1±2.3 | 0.822 |
| CAAP-AF | 3.9±1.5 | 3.7±1.4 | 0.101 |
| MBLATER | 1.2±0.9 | 1.2±0.8 | 0.961 |
| Procedure duration (min) | 84.2±32.3 | 87.7±35 | 0.279 |
| LA dwell time (min) | 59.5±28.8 | 64.2±31.5 | 0.112 |
| Fluoroscopy time (min) | 13.2±5.9 | 13.6±6.2 | 0.557 |
| Radiation dose (mGray) | 282.2±187.2 | 295.9±202.8 | 0.469 |
| Number of PV with real-time recording of PV electrogram | 2.6±1.1 | 2.6±1.1 | 0.834 |
| TTI Score | 1.8±1.2 | 1.7±1.2 | 0.701 |
| Temp_30_ Score | 2.7±1.2 | 2.6±1.2 | 0.864 |
| Temp_60_ Score | 2.6±1.2 | 2.7±1.1 | 0.792 |
| Temp_nadir_ Score | 2.5±1.2 | 2.6±1.2 | 0.487 |
| Number of unsuccessful freezes | 1.7±1.2 | 1.7±1.2 | 0.939 |

Supplementary table 2: Result of the stepwise regression analysis in a forward approach for variable selection

Abbreviation: HR: hazard ration; CI: confidence interval.

| Variable | HR | 95%CI | P value |
| --- | --- | --- | --- |
| Age (year) | 1.011 | 0.989-1.033 | 0.338 |
| Sex (Male) | 0.869 | 0.581-1.300 | 0.495 |
| Body mass index (kg/m2) | 0.982 | 0.915-1.053 | 0.607 |
| Months since first AF diagnosis | 1.003 | 0.998-1.008 | 0.188 |
| Persistent AF | 1.210 | 0.769-1.905 | 0.409 |
| Hypertension | 0.801 | 0.527-1.217 | 0.299 |
| Diabetes mellitus | 0.608 | 0.321-1.151 | 0.126 |
| Coronary artery disease | 0.644 | 0.334-1.240 | 0.188 |
| Previous Stroke/TIA | 0.458 | 0.182-1.150 | 0.096 |
| Left atrial diameter | 1.025 | 0.965-1.087 | 0.424 |
| LVEF | 1.008 | 0.970-1.048 | 0.688 |
| Left atrial volume | 1.005 | 0.999-1.010 | 0.094 |
| TTI Score | 0.499 | 0.403-0.617 | <0.001 |
| Temp_30_ Score | 1.156 | 0.939-1.424 | 0.173 |
| Temp_60_ Score | 0.817 | 0.642-1.039 | 0.099 |
| Temp_nadir_ Score | 0.789 | 0.645-0.966 | 0.022 |
| Number of unsuccessful freezes | 1.328 | 1.125-1.568 | 0.001 |
